# The Diagnostic Value of Clinical Symptoms in Women and Men Presenting with Chest Pain at the Emergency Department, a Prospective Cohort Study

**DOI:** 10.1371/journal.pone.0116431

**Published:** 2015-01-15

**Authors:** Manon G. van der Meer, Barbra E. Backus, Yolanda van der Graaf, Maarten J. Cramer, Yolande Appelman, Pieter A. Doevendans, A. Jacob Six, Hendrik M. Nathoe

**Affiliations:** 1 Department of Cardiology, University Medical Center Utrecht, Utrecht, the Netherlands; 2 Julius Center for Health Sciences and Primary Care, University Medical Center Utrecht, Utrecht, the Netherlands; 3 Department of Cardiology, Zuwe Hofpoort hospital, Woerden, the Netherlands; S.G.Battista Hospital, ITALY

## Abstract

**Background:**

Previous studies suggested that diagnosing coronary artery disease (CAD) is more difficult in women than in men. Studies investigating the predictive value of clinical signs and symptoms and compare its combined diagnostic value between women and men are lacking.

**Methodology:**

Data from a large multicenter prospective study was used. Patients admitted to the emergency department (ED) with chest pain but without ST-elevation were eligible. The endpoint was proven CAD, defined as a significant stenosis at angiography or the diagnosis of a non-ST-elevation myocardial infarction or cardiovascular death within six weeks after presentation at the ED. Twelve clinical symptoms and seven cardiovascular risk factors were collected. Potential predictors of CAD with a p-value <0.15 in the univariable analysis were included in a multivariable model. The diagnostic value of clinical symptoms and cardiovascular risk factors was quantified in women and men separately and areas under the curve (AUC) were compared between sexes.

**Results:**

A total of 2433 patients were included. We excluded 102 patients (4%) with either an incomplete follow up or ST-elevation. Of the remaining 2331 patients 43% (1003) were women. CAD was present in 111 (11%) women and 278 (21%) men. In women 11 out of 12 and in men 10 out of 12 clinical symptoms were univariably associated with CAD. The AUC of symptoms alone was 0.74 (95%CI: 0.69-0.79) in women and 0.71 (95%CI: 0.68-0.75) in men and increased to respectively 0.79 (95%CI: 0.74-0.83) in women versus 0.75 (95%CI: 0.72-0.78) in men after adding cardiovascular risk factors. The AUCs of women and men were not significantly different (p-value symptoms alone: 0.45, after adding cardiovascular risk factors: 0.11).

**Conclusion:**

The diagnostic value of clinical symptoms and cardiovascular risk factors for the diagnosis of CAD in chest pain patients presenting on the ED was high in women and men. No significant differences were found between sexes.

## Introduction

Chest pain is the second most common emergency department (ED) presenting complaint and can be an indicator of coronary artery disease (CAD).[1] In patients presenting with chest pain at the ED a combination of diagnostic tests including patient’s symptoms, electrocardiography (ECG) and troponin is routinely used to diagnose CAD.[2, 3] The diagnostic value of symptoms is particularly important in patients without suggestive ST-segment changes and/ or diagnostic troponin rise and fall.[4, 5] Over 4% of patients with CAD are not recognized at the ED, leading to an increased mortality.[6]

Recently there is growing interest for differences in clinical presentation of women and men with CAD. Previous studies suggested that diagnosing CAD based on symptoms would be more difficult in women than in men.[7–11] Women with CAD appeared to have an atypical clinical presentation compared to men, leading to misdiagnosis and suboptimal treatment.[7–10, 12] Importantly, however, most studies only compared symptoms in women and men with an established diagnosis of CAD. But the crucial unanswered clinical question is which clinical signs and symptoms are associated with CAD in women and men suspected of CAD and whether the combined diagnostic value differs between sexes.

To clarify this issue we examined the predictive value of signs and symptoms and quantified its diagnostic value in women and men visiting the ED with chest pain in a large prospective multicenter study.

## Methods

### Study population

Data from “The prospective validation of the HEART score” study were used.[13] This study was performed at ten hospitals in the Netherlands between 2008 and 2009. Any patient admitted to the (cardiac) ED with chest pain was eligible. The ethics committees of all participating hospitals approved the study and waived informed consent because all patients received standard medical care and the data was analysed anonymously. We excluded patients with a ST-elevation myocardial infarction (STEMI). Moreover, according to current guidelines, patients with a STEMI were directly referred to the catheterization laboratory.[14]

During admission of the patient at the ED, the residents filled in questions about the clinical symptoms, cardiovascular risk factors and past medical history in a structured Case Report Form.

An extensive standard list of 12 clinical symptoms based on common practice and previous research was studied including 7 chest pain symptoms (“oppressive chest pain”, “pain located in the sternal region”, “radiation to jaw/ arm/ shoulder”, “pain started during exercise”, “pain diminished on nitrates”, “same chest pain in last weeks”, “same pain as previous angina pectoris”) and 5 non-chest pain symptoms (“palpitations”, “pulmonary complaints”, “nausea/ vomiting”, “diaphoresis”, “dizziness/ syncope”).[15, 16] On top of that we collected the classical cardiovascular risk factors: age, diabetes, hypertension, dyslipidaemia, current smoking, family history of cardiovascular disease, and medical history of cardiovascular disease. All patients received usual care and the decision for any additional diagnostic tests was left at the discretion of the treating physician.

### Follow-up

Follow up data were retrieved from electronic patient records. In a few cases when data were not available from hospital records, the patient or general practitioner was contacted. Patients were excluded from the analysis in case of an incomplete follow-up not reaching the pre-defined time span of 6 weeks.

### CAD

CAD was considered proven 1) in case of a significant stenosis at angiography requiring percutaneous coronary intervention (PCI)/coronary artery bypass grafting (CABG) or medical treatment within six weeks after presentation at the ED, 2) in patients without angiography, CAD was considered proven in case of a definite diagnosis of a Non-ST-elevation myocardial infarction (NSTEMI) or cardiovascular death within six weeks. NSTEMI was diagnosed using the universal consensus definition.[17] All endpoints were adjudicated by an independent event committee.

### Statistical analyses

Patients were stratified by gender. The cardiovascular risk factors and clinical symptoms were expressed as mean ± standard deviation for continuous variables and as numbers (percentages) for categorical variables. The presence or absence of symptomatic atherosclerotic disease in the medical history, such as myocardial infarction and stroke, peripheral arterial disease and revascularisation procedures were combined into the variable past medical cardiovascular history. The use of different types of antithrombotic medication was combined in one variable. We combined four symptoms fitting a pulmonary origin of the chest pain in the variable “pulmonary complaints”(dyspnoea, coughing, fever and breathing-dependent pain).

We first tested the association between each clinical symptom or baseline characteristic and the presence or absence of CAD using univariable analysis, meaning chi-square in categorical variables and T-test in continuous variables. All candidate predictors with a p-value < 0.15, based on Akaike’s Information Criterion, were included in a multivariable logistic regression model.[18] The first multivariable diagnostic model included only clinical symptoms (model 1). Subsequently cardiovascular risk factors were added to the first diagnostic model (model 2). The ability of the two diagnostic models to discriminate between patients with and without CAD was estimated by the area under the curve (AUC) with 95% confidence intervals (CI), separately in women and men. To compare the obtained AUC of women and men from both models we used bootstrapping by the roc.test from Rpackage “pROC”. All authors had full access to all data.

### Subgroup analyses

As clinical symptoms are most important in patients without typical ECG changes or an elevated first troponin we repeated the analyses in this subgroup of patients. Typical ECG changes were considered present in case of ≥ 1mm ST-segment depression in two continuous leads or elevations or negative T waves in absence of a bundle branch block, left ventricular hypertrophy, or the use of digoxin. Cut off points of Troponin T or I were according to local lab standards and reference values. The majority of the women included were older than 50 years suggesting that they were postmenopausal. Previous studies showed that premenopausal women experienced different clinical symptoms than postmenopausal women.[19, 20] Therefore we repeated the analyses without women younger than 50 years of age.

## Results

**Figure 1:**
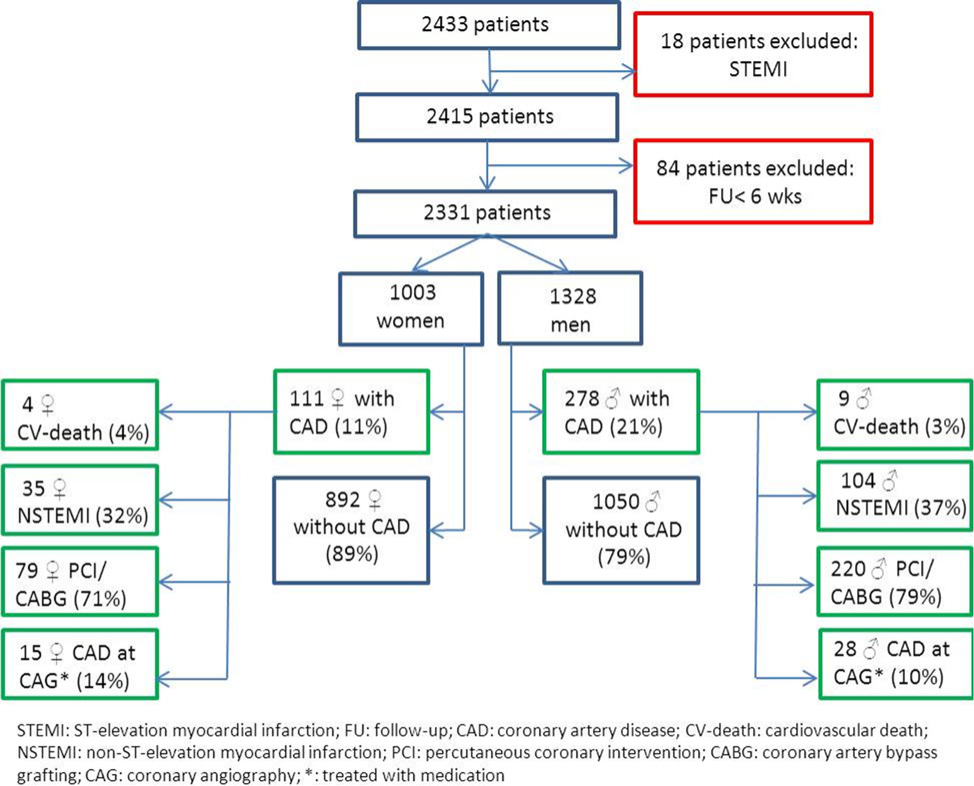
Flowchart.

A total of 2433 patients were included in “The prospective validation of the HEART score” study.[13] We excluded 102 patients (4%) since their follow up did not reach the time span of 6 weeks or they appeared to have a STEMI (Fig. 1). We analyzed the remaining 2331 patients, of whom 43% (1003) were women.

### Baseline characteristics

Women were at average 3 years older than men (62 years versus 59 years). More men than women had a medical history of cardiovascular disease (Table 1). The prevalence of diabetes was comparable between women and men. Compared to women, men were more often smokers and more men had dyslipidemia. The majority of patients experienced “oppressive chest pain”, namely 68% of women and 71% of men. More women than men had accompanying symptoms such as “radiation to jaw/arm/schoulder”, “nausea/ vomiting”, “palpitations” and “dizziness/syncope”. Women experienced more “pain located in the sternal region” while more men had “recognizable pain to previous episode of angina pectoris”.

**Table 1:** Baseline characteristics of women and men (n = 2331).

	**Women**	**Men**	**p-value**
	**n(%)**	**n(%)**	
	**1003 (43)**	**1328 (57)**	
Age in years (SD)	62 ± 16	59 ± 15	<0.01
Cardiovascular risk factors:			
Diabetes Mellitus	180 (18)	262 (20)	0.28
Hypertension	456 (46)	559 (42)	0.10
Dyslipidaemia	329 (33)	506 (38)	0.01
Smoking	302 (30)	455 (34)	0.03
Family history of CV disease	369 (37)	474 (36)	0.59
Past medical cardiovascular history*:	281 (28)	609 (46)	<0.01
Myocardial infarction	102 (10)	271 (20)	<0.01
CABG	57 (6)	182 (14)	<0.01
PCI	145 (15)	359 (27)	<0.01
CVA	42 (4)	68 (5)	0.29
PAD	47 (5)	63 (5)	0.95
Clinical symptoms:			
Oppressive chest pain	716 (71)	902 (68)	0.07
Pain located in the sternal region	682 (68)	801 (60)	<0.01
Radiation to jaw/ arm/ shoulder	521 (52)	569 (43)	<0.01
Pain started during exercise	248 (25)	377 (28)	0.05
Pain diminished on nitrates	173 (17)	264 (20)	0.11
Comparable chest pain in last weeks	459 (46)	601 (45)	0.81
Recognizable pain to previous episode of AP	379 (38)	557 (42)	0.04
Palpitations	172 (17)	119 (9)	<0.01
Pulmonary complaints	378 (38)	451 (34)	0.06
Nausea/ vomiting	307 (31)	259 (20)	<0.01
Diaphoresis	311 (31)	420 (32)	0.75
Dizziness/ syncope	170 (17)	184 (14)	0.04

n: number; SD: standard deviation; CV: cardiovascular;

*: combination of CABG, PCI, CVA, PAD; CABG: coronary artery bypass grafting; PCI: percutaneous coronary intervention; CVA: cerebrovascular accident; PAD: peripheral arterial disease; ECG: electrocardiogram; AP: angina pectoris

### CAD

In total 391 patients, of whom 111 women (11%) and 278 men (21%) were diagnosed with CAD within 6 weeks after the initial presentation at the ED. Among the patients with CAD 13 patients died a cardiovascular death, 139 developed MI, 237 underwent PCI, 66 received CABG and 43 patients had significant CAD by angiography treated conservatively (Fig. 1).

### Univariable analysis

**Figure 2:**
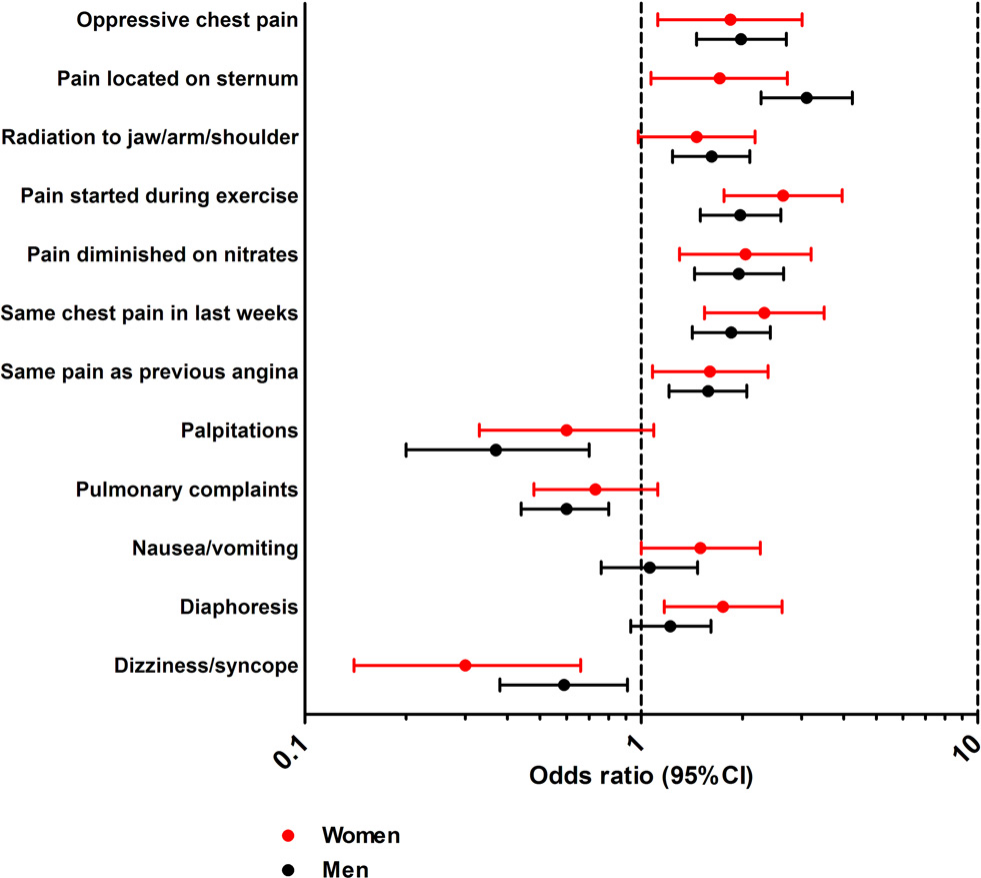
Univariable analysis (odds ratios) of all symptoms in women and men separately.

The univariable association between each clinical symptom and CAD in women and men is visualised in Fig. 2. Overall, there were great similarities in the association of clinical symptoms between women and men. The presence of “dizziness/syncope” was associated with the absence of CAD in women and men. There were a few differences in the magnitude of the association between clinical symptoms and CAD between sexes. For example, “nausea/ vomiting” and “diaphoresis” were positive predictors for CAD in women but not in men. All clinical symptoms except “pulmonary complaints” in women and “nausea/ vomiting” and “diaphoresis” in men had a p-value < 0.15 in the univariable analysis and were added to the multivariable model.

The univariable analysis of cardiovascular risk factors revealed that age, hypertension, dyslipidaemia and a history of cardiovascular disease had a p-value < 0.15 in both sexes. On top of that, in women a fifth cardiovascular risk factor, namely a positive family history of cardiovascular disease, also had a p-value < 0.15.

### Multivariable analysis: clinical symptoms

**Figure 3:**
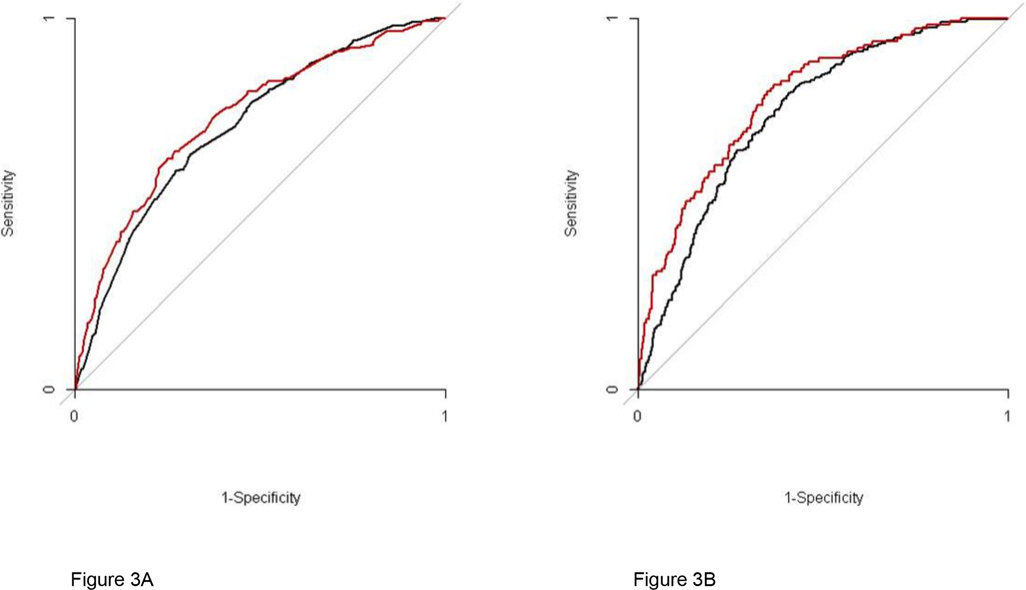
ROC curves of model 1, A, consisting of symptoms. The black line describes the diagnostic value in men and the red line the diagnostic value in women. The AUC in women is not inferior to the AUC in men, p-value 0.45. ROC curves of model 2, B, consisting of symptoms added with baseline characteristics. The black line describes the diagnostic value in men and the red line in women. The AUC in women is not inferior to the AUC in men, p-value 0.11.

In women and men, 8 clinical symptoms remained in this multivariable model (p-value < 0.15, Table 2). The presence of “pain located in the sternal region”, “pain started during exercise”, “pain diminished on nitrates” and “same chest pain in last weeks” were positive predictors for CAD in women and men. “Dizziness/syncope” had a negative predictive value in both sexes. There were some differences between women and men in the first model based on clinical symptoms. “Oppressive chest pain” still qualified as a positive predictor for CAD in women, but in men the p-value exceeded the 0.15 border because other clinical symptoms showed stronger associations. Other positive predictors in women were “nausea/ vomiting” and “diaphoresis”. “Palpitations” and “pulmonary complaints” were negative predictors in men, but had no predictive value in women. The combined diagnostic value of clinical symptoms for the presence of CAD, expressed by the AUC, was 0.74 (95%CI: 0.69–0.79) in women and 0.71 (95%CI: 0.68–0.75) in men (Fig. 3A). This difference in AUC between women and men was not significantly different (p-value 0.45).

**Table 2:** Association (OR +95% CI) between symptoms and CAD in women and men as estimated by multivariable logistic regression analysis (model 1).

	**Women**	**p-value**	**Men**	**p-value**
	**OR (95% CI)**		**OR (95% CI)**	
***Diagnostic model 1: symptoms***				
*Symptoms with positive predictive value:*				
Oppressive chest pain	1.66 (0.99–2.78)	0.05	--	
Pain located in the sternal region	1.50 (0.92–2.43)	0.11	2.78 (2.02–3.84)	<0.01
Radiation to jaw/arm/ shoulder	--		1.56 (1.18–2.07)	<0.01
Pain started during exercise	2.27 (1.45–3.55)	<0.01	1.60 (1.18–2.18)	<0.01
Pain diminished on nitrates	1.82 (1.13–2.93)	0.01	1.51 (1.09–2.09)	0.01
Same chest pain in last weeks	1.81 (1.16–2.83)	0.01	1.49 (1.11–2.00)	0.01
Nausea/ vomiting	1.53 (0.97–2.41)	0.07	--	
Diaphoresis	1.71 (1.10–2.66)	0.02	--	
*Symptoms with negative predictive value:*				
Palpitations	--		0.36 (0.19–0.70)	<0.01
Pulmonary complaints	--		0.57 (0.42–0.79)	<0.01
Dizziness/ syncope	0.21 (0.09–0.46)	<0.01	0.70 (0.45–1.11)	0.13
**AUC**	**0.74 (0.69–0.79)**		**0.71 (0.68–0.75)**	

Only variables from the univariable analysis with a p-value < 0.15 (see table 1 and 2) were included in the multivariable analysis. AUC (area under the curve) was calculated using variables with a p-value <0.15 from the multivariable analysis. The presence of symptoms with a negative predictive value was associated with not having CAD.

### Multivariable analysis: cardiovascular risk factors additional to clinical symptoms

After adding cardiovascular risk factors to the multivariable model age and a history of cardiovascular disease remained positive predictors in women and men (Table 3). In women a positive family history of cardiovascular disease was also associated with CAD as was dyslipidemia in men. In both sexes one clinical symptom lost its predictive value, namely “pain located in the sternal region” in women and “dizziness/syncope” in men (p-value>0.15). After adding the cardiovascular risk factors to the clinical symptoms the AUC of the model increased to 0.79 (95%CI: 0.74–0.83) in women and 0.75 (95%CI: 0.72–0.78) in men (Fig. 3B). The difference in AUC between women and men in model 2 was also not significantly different (p-value 0.11).

**Table 3:** Association (OR +95% CI) between symptoms/cardiovascular risk factors and CAD in women and men as estimated by multivariable logistic regression analysis (model 2).

	**Women**	**p-value**	**Men**	**p-value**
	**OR (95% CI)**		**OR (95% CI)**	
***Diagnostic model 2: clinical symptoms that remained in the model after adding cardiovascular risk factors***				
*Symptoms with positive predictive value:*				
Oppressive chest pain	1.80 (1.06–3.06)	0.03	--	
Pain located in the sternal region	--		2.63 (1.90–3.65)	<0.01
Radiation to jaw/arm/ shoulder	--		1.60 (1.21–2.13)	<0.01
Pain started during exercise	2.34 (1.46–3.75)	<0.01	1.57 (1.15–2.15)	<0.01
Pain diminished on nitrates	1.51 (0.92–2.47)	0.10	1.32 (0.94–1.84)	0.11
Same chest pain in last weeks	1.57 (0.99–2.50)	0.06	1.41 (1.05–1.91)	0.02
Nausea/ vomiting	1.77 (1.11–2.83)	0.02	--	
Diaphoresis	1.78 (1.12–2.82)	0.01	--	
*Symptoms with negative predictive value:*				
Palpitations	--		0.39 (0.20–0.76)	0.01
Pulmonary complaints	--		0.52 (0.38–0.72)	<0.01
Dizziness/ syncope	0.21 (0.09–0.48)	<0.01	--	
***Cardiovascular risk factors***				
Dyslipidaemia	--		1.56 (1.16–2.09)	<0.01
Family history	2.45 (1.54–3.89)	<0.01	--	
Medical history of CVD	1.47 (0.94–2.31)	0.09	1.37 (1.00–1.89)	0.05
Age	1.05 (1.03–1.07)	<0.01	1.03 (1.02–1.05)	<0.01
**AUC**	**0.79 (0.74–0.83)**		**0.75 (0.72–0.78)**	

Only variables from the univariable analysis with a p-value < 0.15 (see table 1 and 2) were included in the multivariable analysis). AUC (area under the curve) was calculated using variables with a p-value <0.15 from the multivariable analysis. The presence of symptoms with a negative predictive value was associated with not having CAD.

### Subgroup analyses

In the subgroup analysis of patients without typical ECG changes or an elevated first Troponin 1698 patients (928 men and 770 women) were included. The area under curve (AUC) of the first model (including clinical symptoms) was 0.72 (95%CI: 0.67–0.78) in men and 0.79 (95%CI: 0.72–0.86) in women. The second model (after adding baseline characteristics) presented comparable results: AUC in men 0.76 (95%CI: 0.71–0.81) and in women 0.84 (0.78–0.89). The AUC of both models differed in favour of women although this difference didn’t reach statistical significance (p-value first model 0.11, second model 0.06). After excluding women younger than 50 years of age 754 women remained in the analyses. The AUC of model 1 (including clinical symptoms) was 0.72 (95%CI: 0.66–0.77) and of model 2 (after adding baseline characteristics) was 0.74 (95%CI: 0.69–0.79). When comparing these AUCs to the AUCs of all men no significant differences were found (p-value model 1: 0.82, model 2: 0.89).

## Discussion

The most important finding was that the diagnostic value of clinical symptoms and risk factors for the prediction of CAD in chest pain patients presenting on the ED was good and not different between women and men. To our knowledge, the quantification of the diagnostic value of clinical symptoms in chest pain patients and its direct comparison between sexes has not been reported before. Our findings in the univariable analysis were concordant with three analyses of chest pain characteristics in patients visiting the ED with chest pain.[21–23] One of these studies also performed a multivariable analysis but in both sexes a minority of the clinical symptoms remained in the multivariable model. Only the AUC of men was published which was poor (0.65). Possibly these results can be explained by the small study groups (246 women, 276 men). [22]

We have closed the existing gap from these previous analyses by adding a multivariable analysis in a large study group and, most importantly, by further quantifying and comparing the diagnostic value of clinical symptoms between sexes.

The diagnostic value of symptoms alone was 0.74 in women and 0.71 in men, indicating that a correct diagnosis of CAD can be achieved in 74% in women and 71% in men by taking the history using a standard set of questions. We added cardiovascular risk factors to the first model since these risk factors are part of risk stratification in patients with chest pain as shown by most risk scores, such as HEART, Framingham and TIMI.[24, 25] After including the cardiovascular risk factors the diagnostic value improved to 0.79 in women and 0.75 in men.

Previous studies showed that more than 80% of patients with symptoms suspected of cardiac ischemia visiting the ED do not have diagnostic changes on the ECG.[13, 26, 27] In addition, in chest pain patients with a negative Troponin the adverse event rate is still 5–9%.[28, 29] Thus a major diagnostic dilemma exists in patients with suspected ischemic symptoms, but normal ECG and Troponin at the ED. Therefore, our research question concerned the diagnostic value of clinical symptoms in patients presenting on the ED with chest pain without taking the ECG or Troponin levels into account. However as clinical symptoms are most important in patients without typical ECG changes or an elevated first troponin we repeated the analyses in this subgroup of patients and the results remained comparable.

Despite the higher age of women, the prevalence of CAD was significantly lower in women (11%) than in men (21%), which is in agreement with previous reports.[21, 30, 31] Since the majority of women was 50 years or older we repeated the analyses without the younger women as previous studies suggested that the clinical presentation could be different in younger women.[19, 20]

“Oppressive chest pain”, often described as the most typical symptom of angina pectoris, was as prevalent in women as in men. In the univariable analysis the predictive value of “oppressive chest pain” was also comparable between sexes but in the multivariable analysis it lost its predictive value in men while it remained the second strongest predictor of CAD in women. This can be explained by other clinical symptoms, closely associated with the presence of “oppressive chest pain”, with a stronger association with CAD in men.

Previous studies frequently compared clinical symptoms between women and men who were already diagnosed with CAD.[12, 19, 32, 33] As the study population and research question are different from our study no comparison about the results can be made since in our study the presence of signs and symptoms was the starting point.

### Strengths and limitations

Our study is a large multicenter prospective study making it possible to extrapolate our results to all patients presenting at the ED with chest pain. The thorough follow-up led to a low exclusion rate of 4%. Furthermore, the diagnosis of CAD was not only obtained at the ED but also at 6 weeks follow-up. On top of that, all endpoints were adjudicated by an independent event committee. A limitation of the study is that even though the results are interesting for patients consulting general practitioners (GP), our results cannot be extrapolated to these patients since our study population comprised only patients that presented at the ED. Two analyses from the primary care setting were however concordant with our findings: clinical symptoms of women and men presenting with acute chest pain at the GP’s attention were largely similar.[34, 35] Second, ideally all patients in a diagnostic study undergo the same reference test to diagnose the disease of interest.[36] As it is not ethical to perform a coronary angiography in all patients presenting at the ED with chest pain we pragmatically used a combination of clinical diagnoses and treatments as the reference standard. This could lead to differential verification bias as previous studies stated that more men than women undergo coronary angiography.[37] However since this would lead to a higher AUC in men, it seems not to be the case in this study. Third, no conclusion can be drawn about possible underlying microvascular disease as in this study only obstructive CAD was evaluated and no additional imaging was performed. Lastly, no information about chest pain duration was collected while this characteristic could have added value.

### Conclusion

The diagnostic value of clinical symptoms and cardiovascular risk factors for the diagnosis of CAD in chest pain patients presenting on the ED was high in both women and men. No significant differences were found between sexes.
